# Expression of functional neuronal receptor latrophilin 1 in human acute myeloid leukaemia cells

**DOI:** 10.18632/oncotarget.10039

**Published:** 2016-06-14

**Authors:** Vadim V. Sumbayev, Isabel Gonçalves Silva, Jennifer Blackburn, Bernhard F. Gibbs, Inna M. Yasinska, Michelle D. Garrett, Alexander G. Tonevitsky, Yuri A. Ushkaryov

**Affiliations:** ^1^ School of Pharmacy, University of Kent, Chatham, Kent, ME4 4TB, United Kingdom; ^2^ School of Biosciences, University of Kent, Canterbury, Kent, CT2 7NJ, United Kingdom; ^3^ Hertsen Moscow Oncology Research Institute, Branch of The National Medical Research Radiological Center, Ministry of Health of The Russian Federation, 125284, Moscow, Russian Federation

**Keywords:** latrophilin, myeloid leukaemia, mammalian target of rapamycin

## Abstract

Acute myeloid leukaemia (AML) is a blood cancer affecting cells of myeloid lineage. It is characterised by rapid growth of malignant leukocytes that accumulate in the bone marrow and suppress normal haematopoiesis. This systemic disease remains a serious medical burden worldwide. Characterisation of protein antigens specifically expressed by malignant cells, but not by healthy leukocytes, is vital for the diagnostics and targeted treatment of AML. Here we report, for the first time, that the neuronal receptor latrophilin-1 is expressed in human monocytic leukaemia cell lines and in primary human AML cells. However, it is absent in healthy leukocytes. Latrophilin-1 is functional in leukaemia cells tested, and its biosynthesis is controlled through the mammalian target of rapamycin (mTOR), a master regulator of myeloid cell translational pathways. Our findings demonstrate that latrophilin-1 could be considered as a novel biomarker of human AML, which offers potential new avenues for AML diagnosis and treatment.

## INTRODUCTION

Latrophilin-1 (LPHN1) is an adhesion G-protein-coupled receptor, which participates in control of calcium signalling and exocytosis in vertebrate neurons and neuroendocrine cells. LPHN1 is highly expressed in the brain, detected at low levels in some other tissues, and reported as absent from blood [1]. Interestingly, this protein and its homologues appear to be upregulated in malignant cells: LPHN1 is found in non-small cell lung cancer and is implicated in its invasive character [2], while LPHN2 and 3 are upregulated or mutated in several types of metastatic cancer [3–5]. Stimulation of LPHN1 induces intracellular Ca^2+^ mobilisation and thus triggers significant exocytosis of neurotransmitters, which is crucial for normal neuronal function [6–7]. LPHN1 also plays an important role in cell surface interactions (reviewed in [1]). Exocytotic activity is also required for cytokine/growth factor release during myeloid cell haematopoiesis [8–11], especially when myeloid leukocytes undergo malignant transformation. Firstly, the energy resources of leukaemia cells are normally very limited [8–12] and lead to oxygen starvation. Therefore, the bone marrow vasculature plays a pivotal role in the survival of leukaemia cells, which induce angiogenesis by releasing vascular endothelial growth factor (VEGF) and other pro-angiogenic factors. Secondly, leukaemic cells produce and release interleukin 6 (IL-6), which is necessary to support their own survival and proliferation [13]. These processes require efficient exocytosis. We therefore hypothesised that myeloid leukaemia cells may express LPHN1, a receptor that is able to convert cell-surface interactions into intracellular signals leading to activation of exocytosis.

Here we report, for the first time, that LPHN1 expression is clearly detectable at both RNA and protein level in human monocytic leukaemia (ML) cell lines (U937 and THP-1) and in primary human acute myeloid leukaemia (AML) cells. Treatment of all these leukaemia cells with the inflammatory mediator lipopolysaccharide (LPS) further upregulates LPHN1 translation *via* mammalian target of rapamycin (mTOR, a master regulator of myeloid cell translation and growth [14]). When LPHN1 is stimulated by its high-affinity ligand [7], α-latrotoxin (LTX), this significantly increases LPS-induced IL-6 release from leukaemia cell lines and primary cells. In contrast, in healthy primary human leukocytes, LPHN1 expression is not detectable and is not induced by the mTOR activators LPS, SCF or anti-Tim-3. We therefore conclude that LPHN1 is a novel pharmacoproteomic biomarker of human AML that offers new approaches to therapeutic targeting of this disease.

## RESULTS

### LPHN1 expression and activity in human ML cell lines

**Figure 1:**
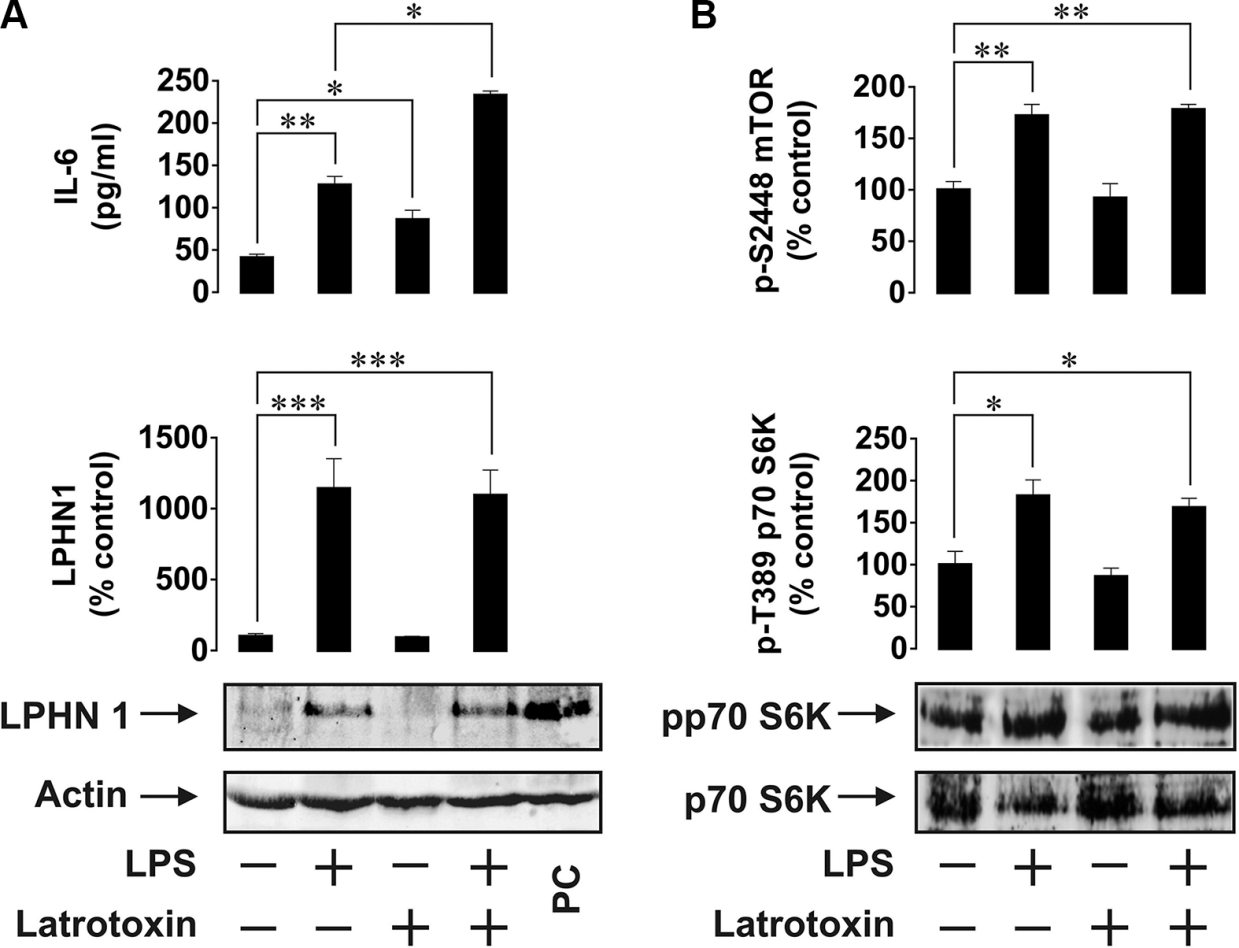
Expression and activity of LPHN1 in U937 human ML cells (**A**) U937 cells were exposed to 1 μg/ml LPS, 500 pM LTX or a combination of these two ligands for 24 h. Following cell lysis, IL-6 release was measured by ELISA, whilst LPHN1 protein levels were analysed by Western blotting. PC; positive control for LPHN1 (mouse brain extract). (**B**) Phospho-S2448 mTOR and phospho-T389 p70 S6K1 were detected using ELISA and Western blotting, respectively. Western blot images show one experiment representative of six, all of which gave similar results. Data are the mean values ± SEM (*n* = 6). *p < 0.05; **p < 0.01; ***p < 0.001.

**Figure 2:**
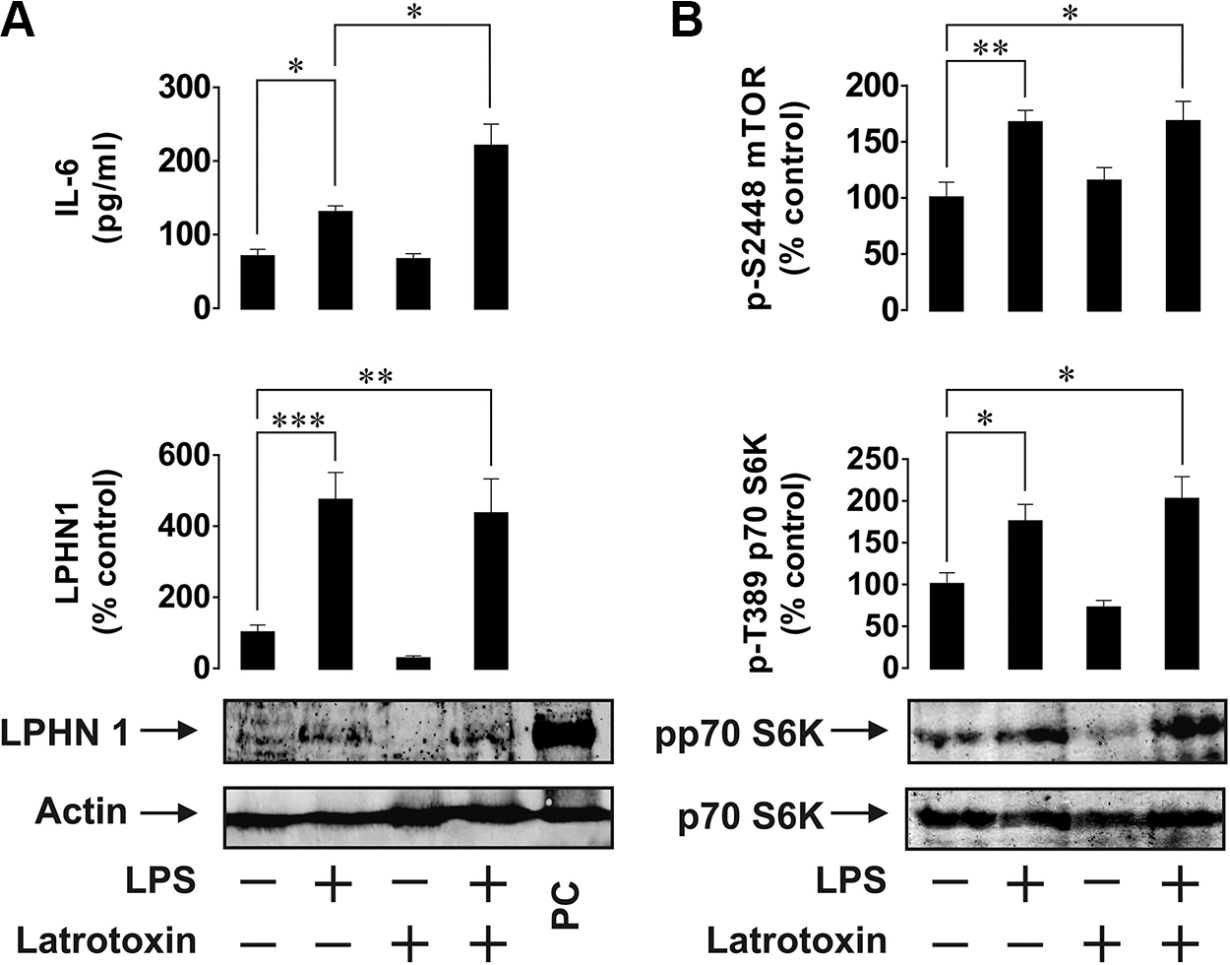
Effects of LPS and LTX on LPHN1 expression, IL-6 exocytosis and mTOR activity in THP-1 human ML cells (**A**) THP-1 cells were exposed to 1 μg/ml LPS, 500 pM LTX or a combination of these two ligands for 24 h. Following cell lysis, LPHN1 protein levels were analysed by Western blot. IL-6 release was measured by ELISA. (**B**) Phospho-S2448 mTOR and phospho-T389 p70 S6K1 were detected using ELISA and Western blot, respectively. Western blot images show one experiment representative of six, which gave similar results. Data are mean values ± SEM (*n* = 6). *p < 0.05; **p < 0.01; ***p < 0.001.

In order to investigate the possibility of LPHN1 expression in human AML cells, we first used human ML cell lines, U937 and THP-1. Cells were stimulated for 24 h with LPS, LTX or a combination of these ligands. LPS is a pathogen-associated molecular pattern shared by Gram-negative bacteria and is recognised by the Toll-like receptor 4 (TLR4), which is expressed by human myeloid cells [13]. LPS was chosen to avoid TLR4 activation by endogenous ligands (such as proteins released after dysfunctionalisation of mitochondria), which themselves induce the expression and release of IL-6 and other important factors required for leukaemia cell survival [15]. Using Western blot analysis, we found that U937 cells constitutively expressed LPHN1 (Figure 1A) and the same pattern was observed in THP-1 cells (Figure 2A). In both U937 and THP-1 cells, LPHN1 expression levels were significantly increased (4-12-fold) by LPS, but not by LTX; when used in combination with LPS, LTX also did not significantly change LPHN1 levels compared to LPS alone (Figures 1A and 2A). However, whilst LTX alone did not stimulate IL-6 release, LTX combined with LPS induced the release of IL-6 that was 2 times greater than for LPS alone in both U937 and THP-1 human ML cells (Figures 1A and 2A).

We also found that LPS, but not LTX, significantly activated the mTOR pathway: LPS augmented by 2-fold the activating phosphorylation of mTOR at S2448 and increased the phosphorylation of its substrate, p70 S6 kinase 1 (p70 S6K1) at position T389. This was clearly observed in both cell lines (Figures 1B and 2B).

**Figure 3:**
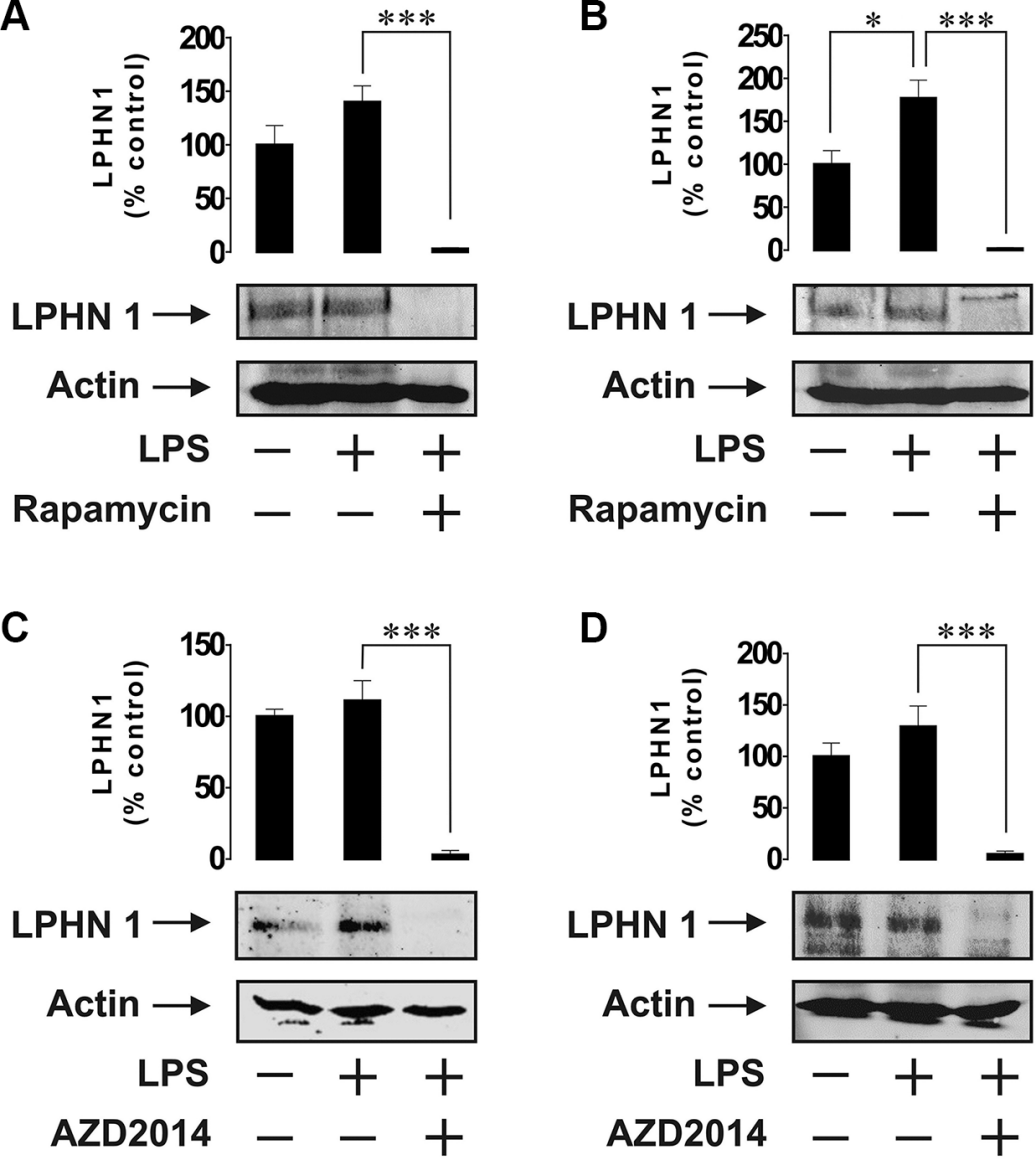
Expression of LPHN1 in U-937 and THP-1 cells depends on mTOR U-937 (**A**, **C**) and THP-1 (**B**, **D**) cells were exposed to 1 μg/ml LPS for 4 h with or without 1 h pre-treatment with 10 μM rapamycin (A, B) or AZD2014 (C, D). LPHN1 expression was then measured using Western blot analysis. Western blot images show one experiment representative of four, which gave similar results. Data are mean values ± SEM (*n* = 4). *p < 0.05; **p < 0.01; ***p < 0.001.

Since mTOR is a master regulator of myeloid cell translational pathways [14], it could be hypothesised that the mTOR pathway is responsible for LPS-induced upregulation of LPHN1 protein levels. We therefore exposed both U937 and THP-1 cells for 4 h to 1 μg/ml LPS with or without 1h pre-treatment with rapamycin (a highly specific mTOR inhibitor). We observed that 4-h exposure to LPS led to a moderate increase in LPHN1 expression in both U937 and THP-1 cells, which was less than the increase induced by 24 h stimulation and more pronounced in THP-1 versus U937 cells. In both cell lines, rapamycin fully blocked the expression of LPHN1 (Figure 3A and 3B). Importantly, rapamycin did not affect the viability of the cells, as verified using MTS cell viability test (data not shown). In order to confirm the role of mTOR signalling in upregulation of LPHN1, we performed a similar experiment using another mTOR inhibitor, AZD2014 [16]. AZD2014 did not affect cell viability as measured by cell viability assay (data not shown); however, in both U937 and THP-1 cells, AZD2014 obliterated expression of LPHN1 protein (Figure 3C and 3D).

### Functional LPHN1 is expressed in human primary AML cells, but not in healthy leukocytes

**Figure 4:**
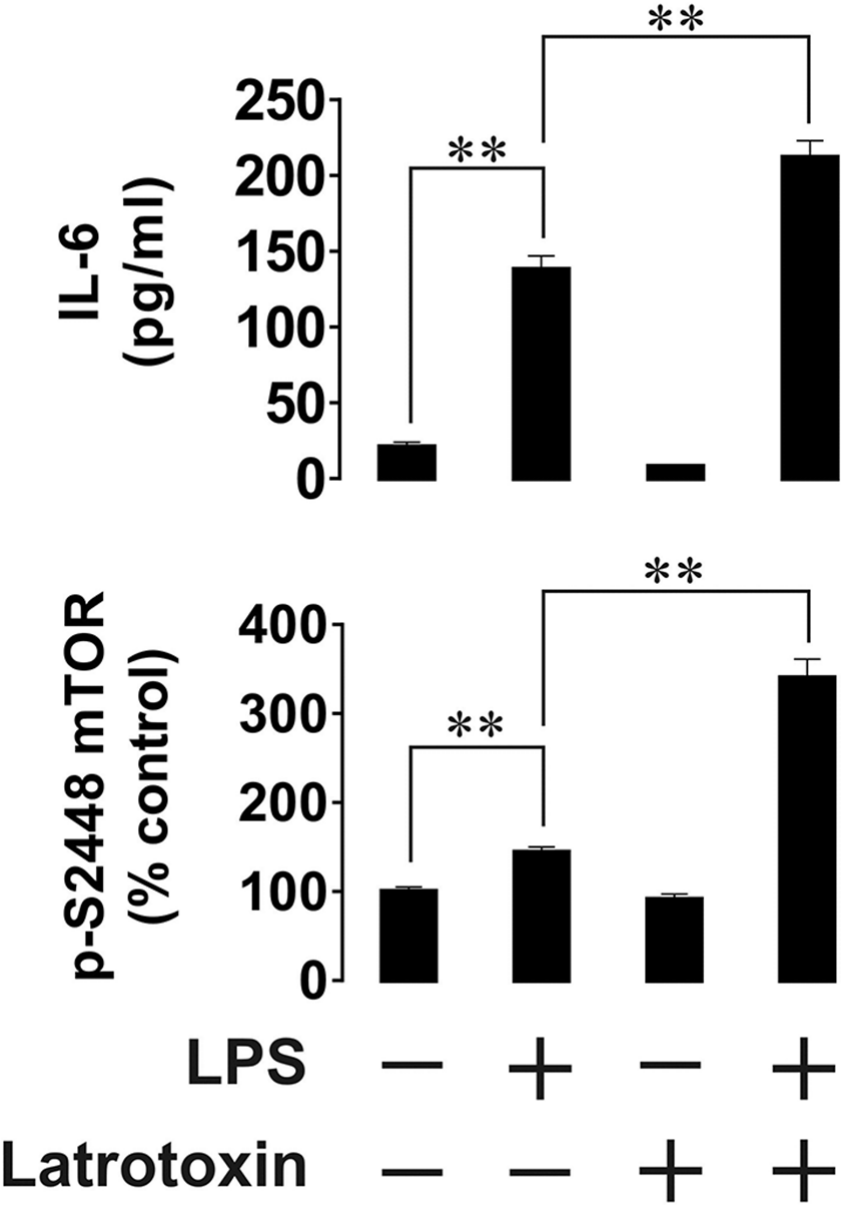
Effects of LPS and LTX on IL-6 exocytosis and mTOR activity in primary human AML cells Primary human AML-PB001F cells were exposed to 1 μg/ml LPS, 500 pM LTX or a combination of these two ligands for 24 h. Following cell lysis, IL-6 release into the medium and mTOR S2448-phosphorylation in cell lysates were measured by ELISA. Data are the mean values ± SEM (*n* = 6). *p < 0.05; **p < 0.01.

**Figure 5:**
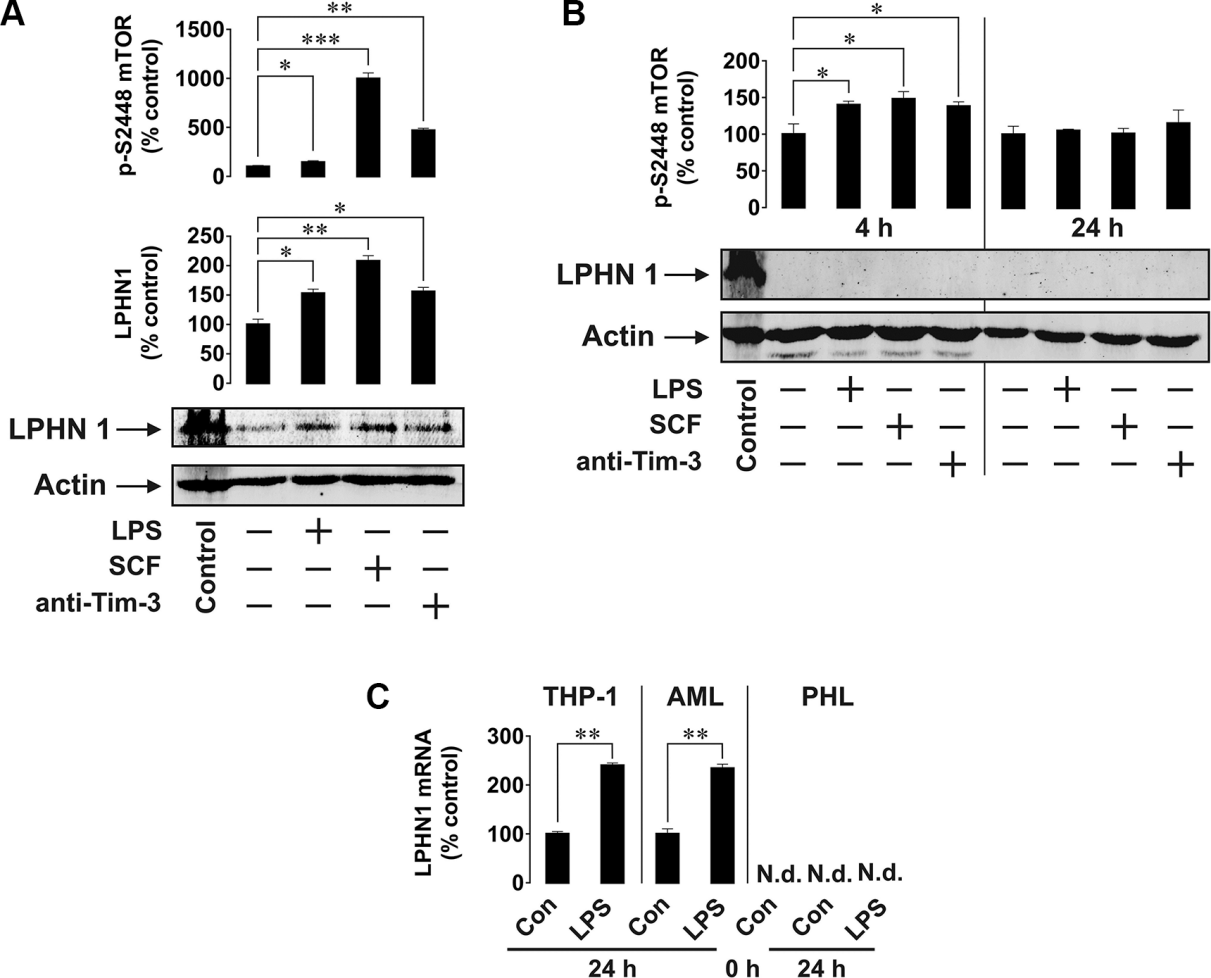
Primary human AML cells but not healthy primary human leukocytes express LPHN1 (**A**, **B**) Primary AML-PB001F cells (A) were exposed for 4 h and healthy human leukocytes (B) for 4 h or 24 h to 1 μg/ml LPS, 0.1 μg/ml SCF or 2 μg/ml anti-Tim-3. Following cell lysis, LPHN1 protein levels were analysed by Western blotting and phospho-S2448 mTOR was quantitated by ELISA. Control, mouse brain extract. (**C**) Quantitative RT-PCR was used to determine the levels of LPHN1 mRNA in THP-1 cells, primary human AML cells and primary healthy human leukocytes (PHL) prior to any treatment (0 h Con), after a 24 h incubation without stimulation (24 h Con), or after a 24 h stimulation with 1 μg/ml LPS (24 h LPS). Data are the mean values ± SEM (*n* = 4). N.d., not detectable; *p < 0.05; **p < 0.01.

Next, we asked whether functional LPHN1 is expressed in primary human AML cells. We exposed AML-PB001F primary human mononuclear blasts for 24 h to LPS, LTX or a combination of these ligands. We found that LPS upregulated both mTOR activation and IL-6 release by these cells. LTX alone was ineffective, but in combination with LPS it significantly increased both mTOR activation and IL-6 exocytosis (Figure 4). In order to determine whether LPHN1 expression levels in AML-PB001F cells were also controlled through the mTOR pathway, we exposed these cells for 4 h to different mTOR activators: LPS, SCF or anti-Tim-3 antibody. We found that each stimulus led to a significant increase in the activating phosphorylation of mTOR (on S2448, Figure 5A), while the SCF-induced effect was stronger due to high levels of Kit receptor expression by these cells [17]. The magnitude of mTOR-dependent effects provoked by LPS, SCF and anti-Tim-3 in human AML cells observed here agrees well with previously published data [17]. Interestingly, all stimuli significantly induced LPHN1 expression, as shown by Western blot analysis (Figure 5A).

Most importantly, when we tested healthy primary human leukocytes, they did not show any LPHN1 expression (even after overexposure of Western blots). Furthermore, LPHN1 was also undetectable following 4 or 24 h exposures of healthy leukocytes to LPS, SCF or anti-Tim-3 antibody (Figure 5B).

To confirm the results based on protein expression, we then conducted quantitative real-time PCR (RT-PCR) to detect and quantify LPHN1 mRNA. These experiments demonstrated that LPHN1 mRNA is detected in THP-1 cells and primary AML cells, but not in healthy primary human leukocytes (Figure 5C). Furthermore, we noted that LPS significantly increased the already high level of LPHN1 mRNA in THP-1 cells and primary AML cells. In contrast, even a 24 h exposure to LPS did not induce LPHN1 mRNA expression in primary healthy human leukocytes (Figure 5C).

Taken together, our findings demonstrate the regulated expression of the functional neuronal receptor LPHN1 in human AML cells, but not in healthy primary human leukocytes. This opens a new direction in research of malignant transformation and survival of cancerous white blood cells and the role of LPHN1 in these processes.

## DISCUSSION

LPHN1 functions as an exocytosis promoter acting through calcium mobilisation/signalling machinery [1, 6, 7]. The presence of regulated exocytosis is one of the main features of neurons and neuroendocrine cells, where this receptor is highly abundant. However, the requirement of exocytosis in malignant cells, in particular myeloid leukocytes, has not yet been investigated and there are no reports regarding a possible role of LPHN1 in leukaemia and pro-leukaemic haematopoiesis.

We therefore considered the possibility of LPHN1 protein expression in human leukaemia cells. Our studies demonstrate that resting ML cells from U937 and THP-1 human cell lines clearly express LPHN1 mRNA and possess detectable amounts of functional LPHN1 protein. We also show that expression of functional LPHN1 is dependent on the mTOR pathway. LTX, a specific LPHN1 agonist [3], significantly increased LPS-induced exocytosis of IL-6 in both cell lines. Since the production of IL-6 protein depends on mTOR [18], and the activity of the mTOR pathway was not upregulated by LTX in either cell line employed, it is evident that LTX triggers IL-6 exocytosis rather than its biosynthesis. Wild type LTX used in our experiments is known to form Ca^2+^-permeable pores in the membrane of cells expressing toxin receptors [6], and such pores could potentially induce LPHN1-independent exocytosis. However, LTX only inserts itself into the membrane after binding the receptor [6], and this interaction always stimulates LPHN1, leading to exocytosis [6, 7]. Indeed, in our experiments, the toxin stimulated secretion in LPHN1-expressing cells only (Figures 1, 2, 4). Furthermore, mutant LTX^N4C^, which does not form pores [7], induced similar exocytosis of IL-6 in THP-1/U-937 cells (data not shown), confirming the active role of LPHN1 in this secretion. Interestingly, LPHN1 expression is completely abolished when the cells are pre-treated with the mTOR inhibitors (rapamycin or AZD2014) before stimulation of U937 or THP-1 cells by LPS (Figure 3). This suggests that the process of LPHN1 expression fully depends on the mTOR pathway. As shown in Figures 1B and 2B, both cell lines demonstrate a background activity of the mTOR pathway, which is further upregulated by LPS, and it is this additional activation of mTOR that brings about the substantial increase in the amount of expressed LPHN1 at 24 h.

**Figure 6:**
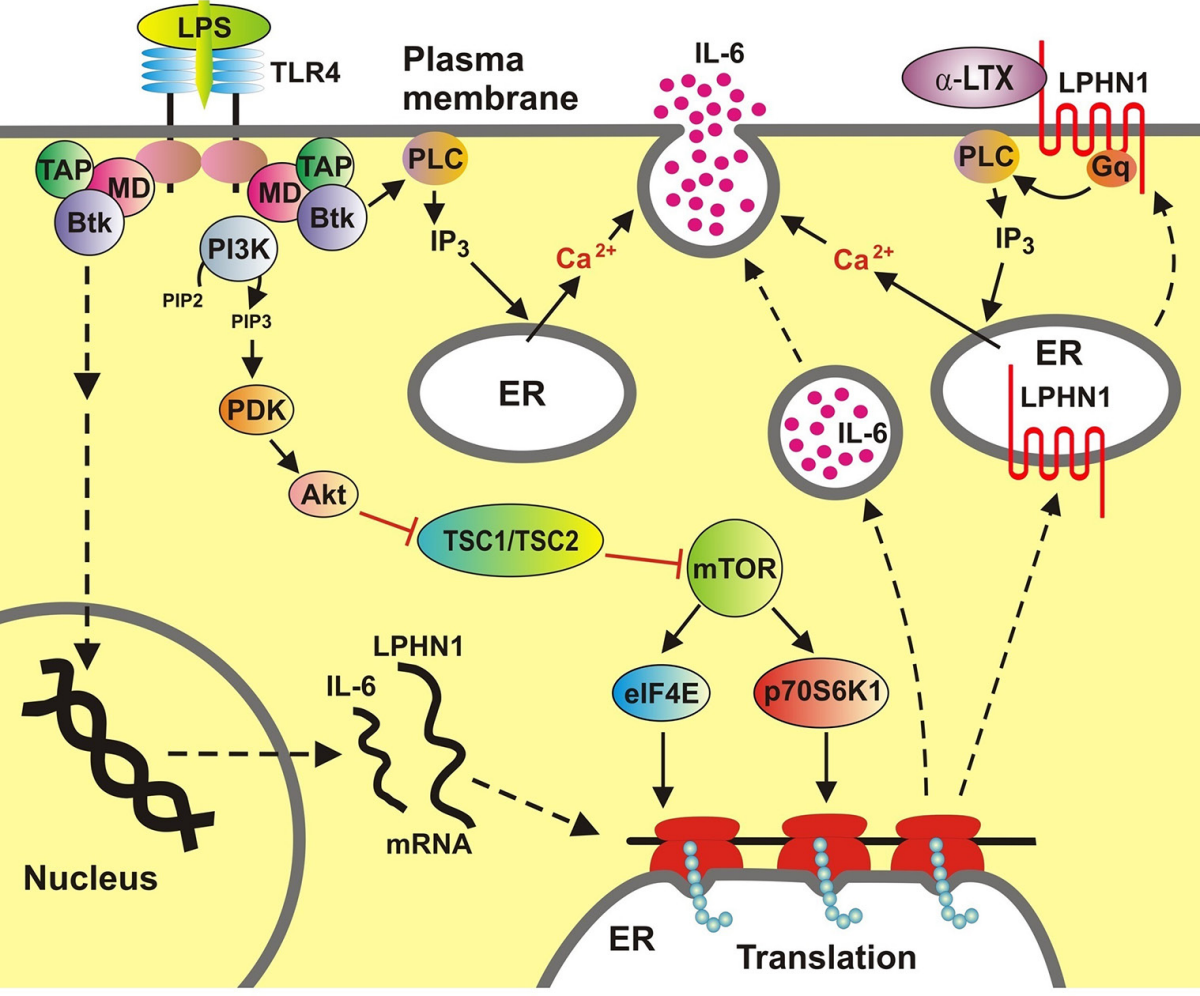
Functional integration of LPHN1 into AML cell signalling machinery LPHN1 transcription and translation is induced in AML cells. LPS upregulates LPHN1 transcription and triggers TLR4-mediated activation of the mTOR pathway, which increases translation of LPHN1 and IL-6. In addition, both LPS (through TLR4 and Btk) and LTX (through LPHN1 and Gq) activate PLC, which produces IP_3_, leading to release of Ca^2+^ from the ER. Increased cytosolic Ca^2+^ triggers exocytosis of IL-6. Abbreviations: Btk, Bruton's tyrosine kinase; MD, myeloid differentiation factor 88; TAP, Toll-like receptor intracellular TIR domain-associated protein; PI-3K, phosphatidylinositol 3-kinase; PDK, phosphatidylinositol-3-phosphate-dependent kinase; IP_3_, inositol trisphosphate; PLC, phospholipase C; Gq, Gαq subunit of a heterotrimeric G protein; TSC1/TSC2, tuberous sclerosis proteins 1 and 2; eIF4E, eukaryotic translation initiation factor 4E; p70S6K1, mTOR-dependent S6 kinase 1; ER, endoplasmic reticulum. Symbols: ←activation; ├ inactivation; dotted lines, indirect process involving multiple steps.

Clearly, ML cell lines differ from malignant leukocytes *in vivo* both in terms of biological activities and protein expression profiles. However, patient-derived primary human AML cells (we used primary AML-PB001F mononuclear blasts obtained from leukaemia patients) do not differ from the two ML cell lines in respect of the easily detectable LPHN1 expression and its upregulation in response to mTOR stimuli (LPS, SCF and anti-Tim-3). Similarly, stimulation of the primary leukaemia cells with LTX leads to a substantial increase in the release of LPS-induced IL-6. On the other hand, unlike in the cell lines, LTX also upregulates LPS-induced mTOR-activating phosphorylation in the primary AML cells. Our observed effect is likely to be the result of low expression of the LPS receptor, TLR4, in the primary AML cells, as reported by their supplier. As a result of the lower TLR4 signalling, only some mTOR will be activated by LPS, while the majority of mTOR will still remain unphosphorylated and therefore subject to further phosphorylation through LPHN1-induced signalling. In cell lines, where the TLR4 expression levels are higher, there may not be sufficient scope for further activation of mTOR by LTX. This finding implies a functional integration of LPHN1 into the AML signalling machinery [13, 19]. Its possible functions are summarised in Figure 6.

One very important finding of this work is that primary healthy human leukocytes do not express any LPHN1. Furthermore, LPHN1 expression is not inducible by exposure of the healthy cells to any of the stimuli that show positive effects in AML cells: LPS, SCF or anti-Tim-3. This clearly demonstrates that the expression of functional LPHN1 occurs specifically in leukaemia cells only. Quantitative RT-PCR experiments further confirm that healthy primary leukocytes do not possess detectable LPHN1 mRNA. This indicates that the change in the expression profile of this important receptor only takes place in leukocytes during or as a result of malignant transformation. The importance of LPHN1 to malignant leukocytes is evident: for their survival, these cells release certain growth factors and cytokines (for example, VEGF and IL-6). This requires not only the presence of efficient exocytotic machinery, but also the means of controlling and stimulating exocytosis, for example, in response to leukocyte cell-surface interactions.

LPNH1 can, therefore, be further considered as a novel biomarker for AML diagnostics. Most existing leukaemia biomarkers are relative: they are expressed in both healthy and malignant cells, but their levels are higher in malignant cells; in contrast, LPHN1 is totally absent from healthy leukocytes and is thus an absolute biomarker of leukaemic cells. Furthermore, LPHN1 could now be explored as a potential novel target for anti-leukaemia therapy and drug delivery. For this purpose, it would be essential to identify a ligand that could bind and activate LPHN1 in leukaemia cells. Such a ligand would not only allow a full characterisation of the malignant transformation pathway, but would also provide a potential therapeutic target(s) to prevent LPHN1-induced exocytosis in leukaemia cells. The ligand of leukaemia LPHN1 could be a soluble factor circulating in blood plasma or it could be expressed on the surface of certain blood, endothelial or bone marrow cells. We previously characterised the natural LPHN1 ligand of nervous tissue (Lasso/teneurin-2) [20]. However, in the current work we were unable to detect Lasso by Western blot in ML cell lines (THP-1 and U-937), primary healthy leukocytes or blood plasma obtained from healthy donors (data not shown). Future research will concentrate on the identification and isolation of peripheral ligand/s of leukaemic LPHN1.

## MATERIALS AND METHODS

### Materials

All cell culture reagents were from Sigma (Suffolk, UK). Microtitre plates were obtained from Nunc (Roskilde, Denmark). LTX and LTX^N4C^ were purified as previously described [6]. Mouse monoclonal antibodies to mTOR and β-actin and rabbit polyclonal antibodies against phospho-S2448 mTOR were from Abcam (Cambridge, UK). Antibodies against phospho-T389 and total p70 S6 kinase 1 (p70 S6K1) were obtained from Cell Signalling Technology (Danvers, MA USA). Goat anti-mouse and goat anti-rabbit fluorescent dye-labelled antibodies were from Li-Cor (Lincoln, Nebraska USA). ELISA-based assay kits for the detection of IL-6, was from R&D Systems (Abingdon, UK). The polyclonal rabbit anti-peptide antibody PAL1 against LPHN1 and polyclonal mouse antibody dmAb against Lasso/teneurin-2 were previously described [20, 21]. Human SCF [22] and anti-Tim-3 monoclonal antibody [23] were a kind gift of Dr. Luca Varani.

### THP-1 and U937 human myeloid cells

THP-1 human myeloid leukaemia monocytic macrophages and U937 human leukaemia monocytes were obtained from the European Collection of Cell Cultures (Salisbury, UK). Cells were cultured in RPMI 1640 media supplemented with 10% foetal calf serum, penicillin (50 IU/ml) and streptomycin sulphate (50 μg/ml).

### Primary human AML cells

Primary human AML mononuclear cells (AML-PB001F, newly diagnosed/untreated) were purchased from AllCells (Alameda, CA, USA) and handled in accordance with manufacturer's instructions. Cells from four different patients were used in reported experiments.

### Primary human leukocytes from healthy donors

Primary human leukocytes were obtained from blood buffy coat (prepared from healthy donors) purchased from the National Health Blood and Transfusion Service (NHSBT, UK) following ethical approval (REC reference: 12/WM/0319) [17, 24]. Mononuclear-rich leukocytes were obtained by Ficoll-density centrifugation according to the manufacturer's (GE Healthcare Life Sciences) protocol. Cell numbers were determined using a haemocytometer and diluted accordingly with HEPES-buffered Tyrode's solution before treatment, as indicated.

### Western blot analysis

The levels of LPHN1, total and phospho-T389 p70 S6K1 as well as Lasso/teneurin 2 were determined by Western blot analysis, as previously described [17, 23]. Fluorescently labelled antibodies (Li-Cor) were used according to the manufacturer's protocol to visualise the proteins of interest using an Odyssey imaging system (Li-Cor). Western blot data were subjected to quantitative analysis using the Odyssey software and values were normalised against respective β-actin bands.

### Quantitative real-time reverse transcription PCR (qRT-PCR)

Total RNA was extracted using the Illustra RNAspin Midi RNA isolation kit (GE Healthcare) and quantified spectroscopically with a Nanodrop 2000^®^ (Thermo Scientific). cDNA was synthesised using Transcriptor First Strand cDNA Synthesis Kit (Roche), which was performed in accordance with the manufacturer's protocol. Relative quantification of LPHN1 mRNA was performed using SYBR Green I Master reaction mix (Roche) and a LightCycler 480 (Roche). The house-keeping gene β-actin was used as a reference gene. The following primers were used at a final concentration of 0.5 μM: LPHN1, 5′-AGCCGCCCCGAGGCCGGAACCTA-3′ and 5′-AGG TTGGCCCCGCTGGCATAGAGGGAGTC-3′; Actin, 5′-T TCGCGGGCGACGATGC-3′ and 5′-GGGGCCACACGC AGCTCATT-3′.

PCR reactions began with incubation at 95°C for 3 min 30 s, then proceeded for 45 cycles of 95°C for 10 s, 60°C for 20 s and 72°C for 10 s. Fluorescence level was detected at 80°C in each cycle. A final elongation step was held at 72°C for 5 min. Raw fluorescence data were analysed using LinRegPCR quantitative PCR data analysis programme [25]. Amplified products were examined on 1.5% agarose gel containing ethidium bromide.

### Detection of phospho-S2448 mTOR in cell lysates by ELISA

Phosphorylation of mTOR was monitored using ELISA assays as recently described [15]. Briefly, ELISA plates were coated with mouse anti-mTOR antibodies and blocked with 2% BSA. Cell lysates were then added to the wells and incubated with agitation at room temperature for 2 h. After washing with TBST buffer, anti-phospho-S2448 mTOR antibody was added and plates were incubated with agitation for 2 h at room temperature. Plates were then washed with TBST buffer and incubated with 1:1000 HRP-labelled goat anti-rabbit IgG in TBST buffer. After washing with TBST, bound secondary antibodies were detected by the peroxidase reaction (ortho-phenylenediamine/H_2_O_2_).

### Detection of exocytosed IL-6

Concentrations of IL-6 released into cell culture media were analysed by ELISA (R&D Systems assay kit) according to the manufacturer's protocol.

### Cell viability assay

Cell viability was analysed using the Promega (Southampton, UK) MTS (3-(4,5-dimethylthiazol-2-yl)-5-(3-carboxymethoxyphenyl)-2-(4-sulfophenyl)-2H-tetrazolium) assay kit according to the manufacturer's protocol.

### Statistical analysis

Each experiment was performed 3–6 times and statistical analysis was conducted using ANOVA test with Bonferroni correction.
